# Preclinical Investigations on Anti-fibrotic Potential of Long-Term Oral Therapy of Sodium Astragalosidate in Animal Models of Cardiac and Renal Fibrosis

**DOI:** 10.1021/acsptsci.3c00264

**Published:** 2024-01-12

**Authors:** Xiao-Yi Chen, Tian-Tian Wang, Qing Shen, Hao Ma, Zhan-Hua Li, Xi-Na Yu, Xiao-Feng Huang, Lin-Sen Qing, Pei Luo

**Affiliations:** ∧ State Key Laboratories for Quality Research in Chinese Medicines, Faculty of Pharmacy, Macau University of Science and Technology, Macau 999078, China; ‡ Chengdu Institute of Biology, Chinese Academy of Sciences, Chengdu 610041, China; § Collaborative Innovation Center of Seafood Deep Processing, Zhejiang Province Joint Key Laboratory of Aquatic Products Processing, Institute of Seafood, Zhejiang Gongshang University, Hangzhou 310012, China; ∥ Institute of Medicinal Biotechnology, Chinese Academy of Medical Sciences, Peking Union Medical College, Beijing 100050, China

**Keywords:** Sodium astragalosidate, cardiac fibrosis, renal fibrosis, TGF-β1, Smads

## Abstract

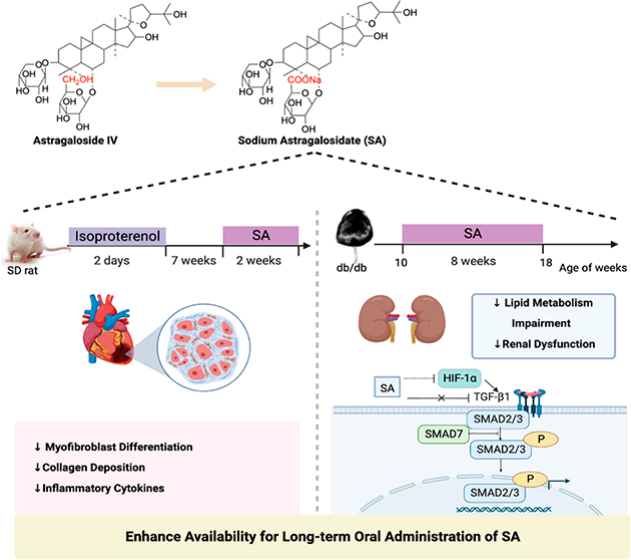

In traditional Chinese medicine, Radix Astragali has played a vital role in treating progressive fibrotic diseases. One of its main active components, astragaloside IV, is a promising anti-fibrotic treatment despite its extremely low bioavailability. Our study aimed to optimize sodium astragalosidate (SA) by salt formation to improve solubility and oral absorption for anti-fibrotic therapy *in vivo*. Isoproterenol-induced myocardial fibrosis rat models and obese BKS-db mice presenting diabetic kidney fibrosis were used in this study. Daily oral administration of SA (20 mg/kg) for 14 days ameliorated cardiac fibrosis by reducing collagen accumulation and fibrosis-related inflammatory signals, including TNF-α, IL-1β, and IL-6. In db/db mice, SA (5,10, and 20 mg/kg per day for 8 weeks) dose-dependently alleviated lipid metabolism impairment and renal dysfunction when administered orally. Furthermore, Western blot and immunohistochemistry analyses demonstrated that SA treatment inhibited renal fibrosis by suppressing TGF-β1/Smads signaling. Taken together, our findings provide the oral-route medication availability of SA, which thus might offer a novel lead compound in preclinical trial-enabling studies for developing a long-term therapy to treat and prevent fibrosis.

Fibrosis is a dysregulated deposition of the extracellular matrix (ECM) that accompanies the progressive destruction of normal tissue. It is the ultimate pathological feature of many chronic diseases, which begins with an abnormal tissue regeneration process, leading to persistent and/or severe tissue damage and cellular stress. Abnormal regeneration in the injured tissue is more common than normal regeneration and is characterized by persistent inflammation, tissue necrosis, activation of myofibroblasts, excessive accumulation of ECM, and ultimately permanent irreversible tissue fibrosis. If its highly progressive, the fibrotic process can eventually lead to organ dysfunction and death, which accounts for approximately 45% of all deaths in Western developed countries.^1^ Fibrosis occurs mainly in various organs, such as the lungs, heart, and kidneys. Of these, cardiac fibrosis is mainly associated with various cardiac diseases, such as chronic heart failure, atrial fibrillation, and cardiac remodeling following acute myocardial infarction.^2^ Renal fibrosis is associated with many forms of chronic kidney disease and impairment of kidney function. Each of these conditions represents an important unmet medical need that requires significant research and clinical studies for the treatment of fibrotic disease. Of the various kinds of administration of medicines, oral therapy is considered to be the most acceptable and cost-effective method in clinical settings.^3^ Although understanding of the pathogenesis and management of fibrotic disease has now increased considerably, few oral drugs specifically target fibrosis in the clinic. In addition, the available commercial oral drugs have serious side effects that limit their clinical use.^4^ Therefore, the search for a new lead compound for developing an oral therapy against fibrosis in clinical is of great importance.

Traditional Chinese medicine (TCM) has been used to treat chronic diseases, particularly fibrosis, for a long time.^5^ In some studies, TCM has been found to yield anti-fibrotic effects by restoring abnormal lipid metabolism. Among TCMs, Radix Astragali, sweet in flavor and warm in nature, has the efficacy of “Fuzheng Guben” and “Yiqi Gubiao”, which are commonly used plants in food and medicine.^6^ Astragaloside IV (AS-IV), as the main pharmacologically active component of Radix Astragali, is used as an indicator compound for the identification of Radix Astragali or Astragali-containing medicines in the Chinese Pharmacopoeia.^7^ In the clinic, it is often used to treat serious complications caused by diabetes, such as kidney damage, myocardial inflammation, and fibrosis.^8^ Some studies have shown that AS-IV could alleviate myocardial damage and fibrosis induced by diabetes in rats by improving the abnormal lipid metabolism.^9^ In recent years, many studies have shown that AS-IV can inhibit glomerular thylakoid over-proliferation via the TGF-β1/Smad/miR-192 signaling pathway, thereby reducing renal fibrosis.^10^ Astragali decoction exerts its protective effect against kidney injury in UUO mice by down-regulating TGF-β/Smads signaling.^11^ Moreover, AS-IV also exerts its anti-fibrotic effects on organs, especially the heart and kidney, by inhibiting ROS, apoptosis, and inflammation pathways.^12^ However, the poor water solubility and bioavailability *in vivo* of AS-IV have limited further development of the drug as an oral therapy in the clinic. Its absolute oral bioavailability is only 7.4% in beagles and 2.2% in rats, respectively.^13−14^ Salt formations were devoted to optimizing sodium astragalosidate (SA) to improve solubility and oral absorption for an anti-fibrotic oral therapy starting from the previously synthesized AS-IV derivative. However, to date, little is known about the anti-fibrotic effect of long-term oral therapy of SA *in vivo*. Therefore, the aim of this study was to investigate the anti-fibrotic effects of long-term oral SA treatment in an attempt to elucidate the molecular mechanisms. This study might provide new insights and basic research to explore the further development of SA as an oral medication for the treatment and prevention of fibrosis.

## Results

### SA Ameliorated ISO-Induced Myocardial Injury and Fibrosis in Rats

**Figure 1:**
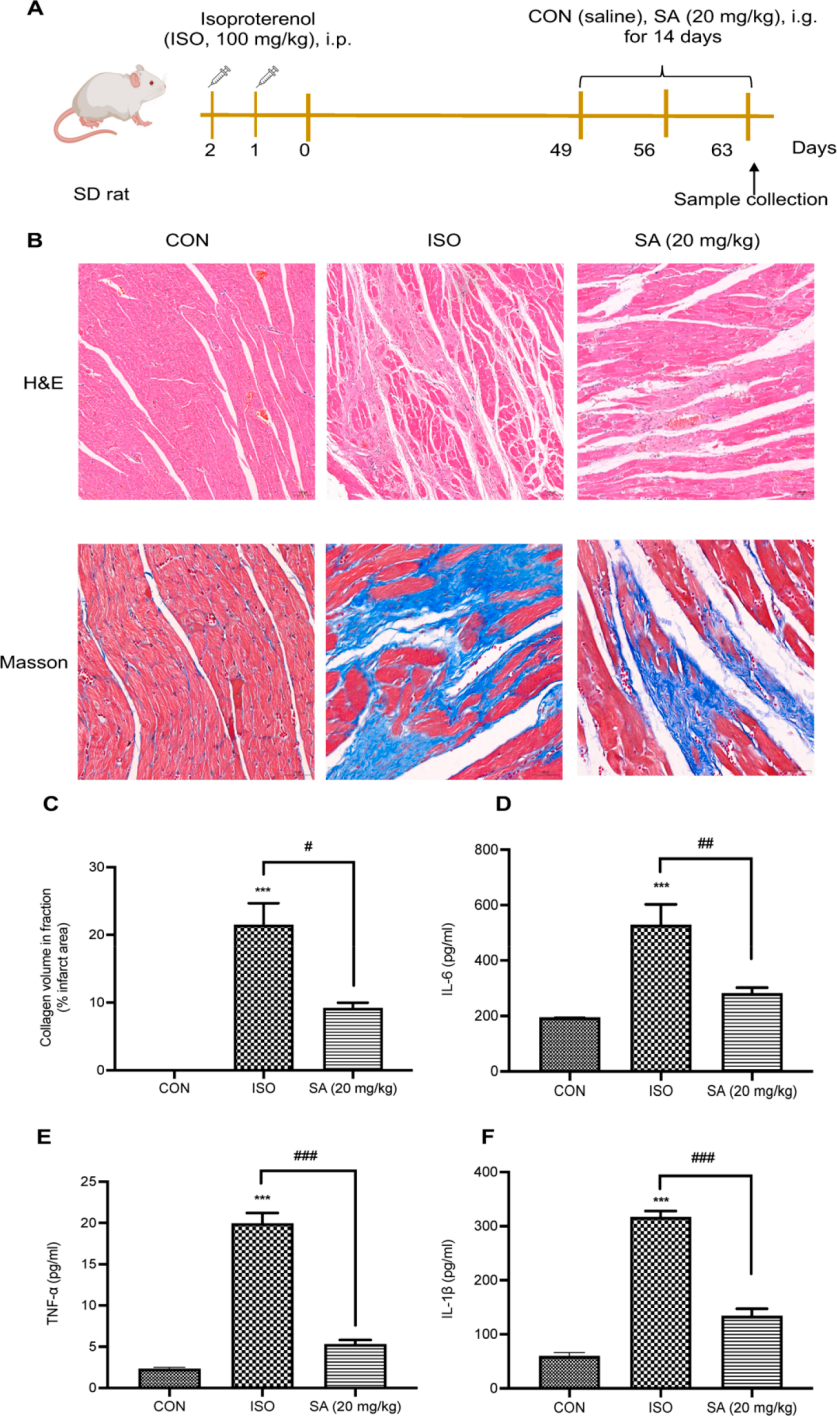
**Effect of SA on ISO-induced myocardial injury and fibrosis rats.** (A) Outline of experimental protocol to evaluate the effect of SA against ISO-induced cardiac fibrosis (*n* = 8). (B) H&E and Masson staining shows the cardiac structural damage of myocardial fibrosis induced by ISO in rats (100× and 400× magnification). (C) Collagen volume fraction quantified from the stained sections. (D–F) Effect of SA on serum levels of IL-6, TNF-α, and IL-1β of myocardial fibrosis in rats. All data are represented as the mean ± SEM. *p < 0.05, **p < 0.01, and ***p < 0.001 compared with the control group; ^#^*p* < 0.05, ^##^*p* < 0.01, and ^###^*p* < 0.001 compared with the ISO group.

We used isoproterenol (ISO), a β-adrenergic receptor agonist, to induce cardiac fibrosis.^15^ As presented in Figure 1B, H&E staining was performed to assess pathological changes in heart tissues. H&E staining in the ISO group showed disturbed myocardial fiber arrangement and myofilament breakage with lysis and necrosis. The histological changes in the heart were significantly ameliorated after oral administration of SA for 14 days. Moreover, Masson’s trichrome staining was used to evaluate the extent of myocardial injury. As shown in Figure 1B,C, massive deposition of collagen protein (blue) accumulated in the disrupted myocardium of the ISO group. The pathological changes described above suggested that the model of myocardial fibrosis was successfully established. However, oral administration of SA for 14 days decreased the ratio of collagen area in myocardial tissues (*P* < 0.01), indicating the anti-fibrotic effect of SA. Inflammation is a key factor in the process of myocardial remodeling. As shown in Figure 1D–F, a significant increase was found in serum levels of IL-6, TNF-α, and IL-1β in the ISO group (*P* < 0.01). As expected, the levels of TNF-α, IL-1β, and IL-6 in serum were significantly decreased after SA treatment. Most importantly, SA (20 mg/kg) treatment reversed the levels of TNF-α and IL-1β in serum to those of the control group. The above data showed that daily oral administration of SA (20 mg/kg) for 14 days ameliorated cardiac fibrosis by reducing fibrosis-related inflammatory signals, including TNF-α, IL-1β, and IL-6.

### SA Inhibited ISO-Induced Fibrotic Gene Expression in Rat Heart Tissues

**Figure 2:**
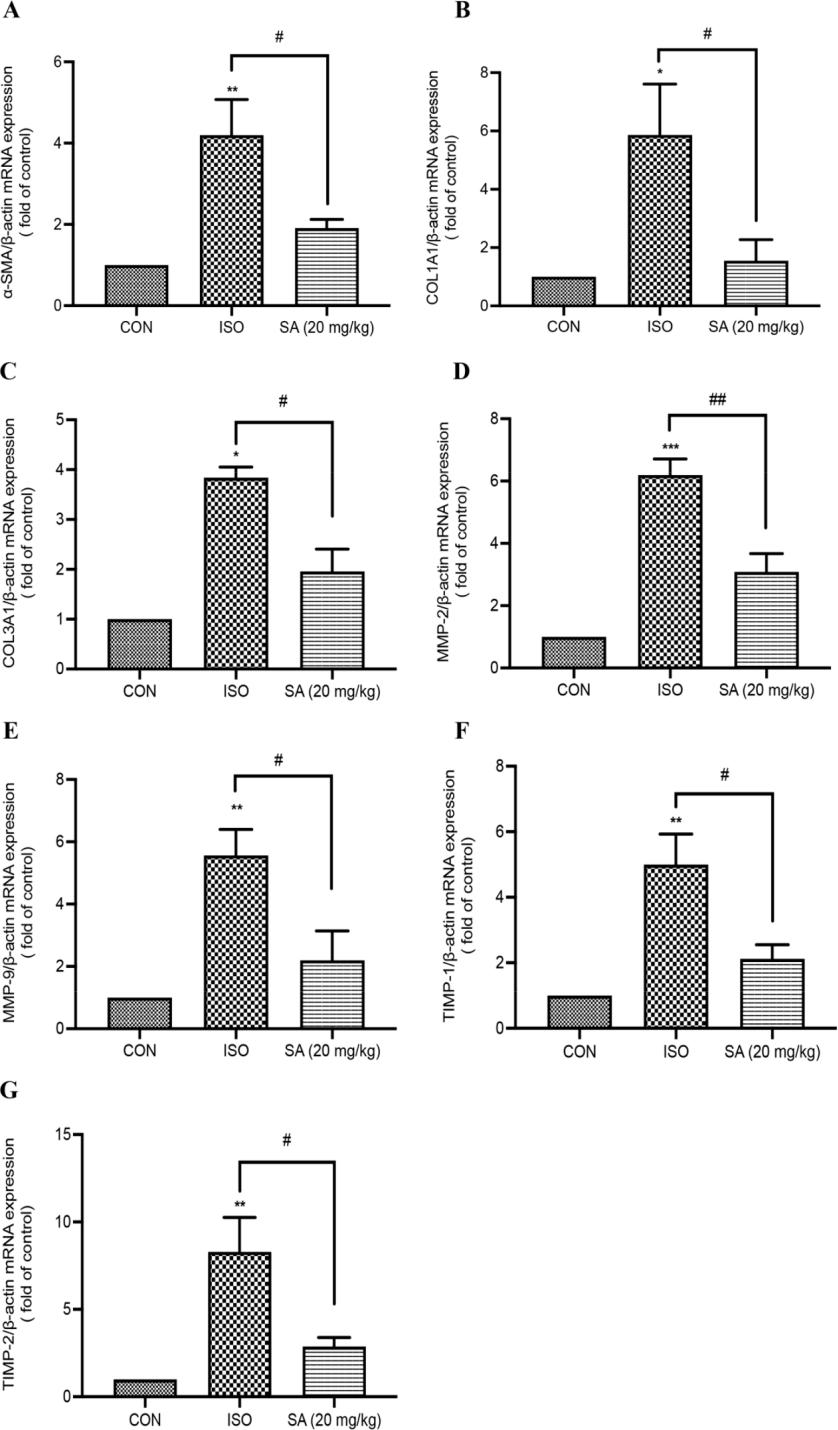
**Effect of SA on ISO-induced fibrotic gene expression in heart tissues.** (A) qRT-PCR analysis of α-SMA and collagen I/III mRNA expression in ISO-induced myocardial fibrosis in rats. (B–G) qRT-PCR analysis of MMP-2/9 and TIMP-1/2 mRNA expression in ISO-induced myocardial fibrosis in rats. All data are represented as the mean ± SEM. *p < 0.05, **p < 0.01, and ***p < 0.001 compared with the control group; ^#^*p* < 0.05, ^##^*p* < 0.01, and ^###^*p* < 0.001 compared with the ISO group.

In addition to alterations in cardiac pathological changes, we also noted changes in fibrotic gene expression after SA treatment in rats. α-SMA, one of the most potent markers in myofibroblast differentiation, can lead to an increase in collagen synthesis.^16^ Increased expression of type I and type III collagen are one of the main phenotypes of cardiac fibrosis.^17^ As shown in Figure 2A–C, the mRNA expression of α-SMA and type I and III collagen in the ISO group was significantly upregulated by using qRT-PCR. Oral administration of SA for 14 days significantly decreased the mRNA expression of α-SMA and type I and III collagen in heart tissues. In addition, a significant effect of SA intervention was found to be the restoration of mRNA expression of type I collagen to the level of the control group. Moreover, matrix metalloproteinases (MMPs) are essential substances for the degradation of ECM. The expected increases were detected in the mRNA expression of MMP-2/9 and TIMP-1/2 in the ISO group (Figure 2D–G). These increases were significantly reversed by SA treatment for 14 days. The above results suggested that SA might regulate collagen synthesis and degradation to alleviate cardiac injury and fibrosis.

### SA Attenuated Lipid Metabolism Impairment in db/db Mice

**Figure 3:**
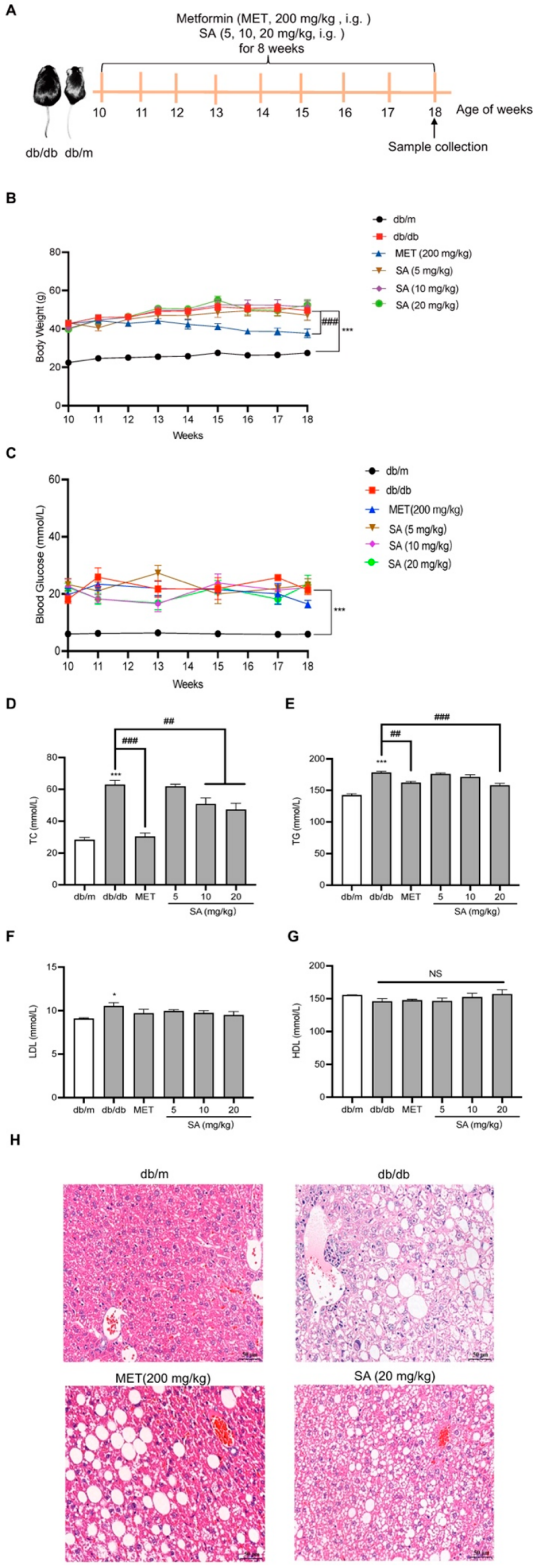
**Oral administration of SA inhibited lipid metabolism impairment in db/db mice.** (A) Outline of experimental protocol to evaluate the effect of SA against renal fibrosis in db/db mice (*n* = 8). (B, C) Effects of SA on body weight and blood glucose in db/db mice (*n* = 8). (D–G) The levels of TC, TG, LDL, and HDL in serum were analyzed in the experiment (*n* = 8). (H) Illustration of H&E staining of the liver tissue in different groups. Magnification 400×. All data are represented as the mean ± SEM. *p < 0.05, **p < 0.01, and ***p < 0.001 compared with the db/m group; ^#^*p* < 0.05, ^##^*p* < 0.01, and ^###^*p* < 0.001 compared with the db/db mice.

The db/db mice presenting diabetic renal fibrosis were also used to explore the anti-fibrotic effects of SA for long-term oral therapies. In our study, the db/db mice were obese when shipped to the laboratory, as their baseline weight was much higher than that of the db/m mice. Unlike db/m mice, db/db mice gained significantly more weight, became progressively more lethargic, responded more slowly, moved less, consumed more food and water, and urinated more for 8 weeks (Table 1). As represented in Figure 3B,C, db/db mice showed higher body weight and blood glucose during the experiment period. A significant decrease was observed in body weight and blood glucose after metformin (MET, 200 mg/kg) treatment. However, there were no significant differences in these parameters after treatment with different doses of SA, suggesting that SA did not affect body weight or blood glucose in db/db mice. Moreover, the levels of total cholesterol (TC), triglycerides (TG), low-density lipoprotein (LDL), and high-density lipoprotein (HDL) in serum were detected at the end of the experiment. As shown in Figure 3D–G, the expression of TC, TG, and LDL in the serum of db/db mice was significantly increased, while the serum level of HDL in db/db mice remained at normal levels throughout the experiment. Oral administration of SA for 8 weeks caused a reduction in TC and TG levels and tended to increase HDL levels in serum, although the difference was not significant compared to db/db mice. In addition, H&E staining of the livers was also used to observe hepatic steatosis (Figure 3H). The db/db mice showed marked steatosis, as evidenced by enlarged hepatocytes containing lipid droplets of different sizes and significantly swollen hepatocytes. Specifically, the effect of SA (20 mg/kg) treatment was noted to restore lipid deposition to that of the MET group. The above results showed that the oral administration of SA for 8 weeks significantly inhibited lipid metabolism impairment in db/db mice.

**Table 1:** Effect of SA on Body Weight, Food Intake, Water Intake, and the Ratio of Feed Efficiency in db/db Mice^a^

	db/m	db/db	MET (200 mg/kg)	SA (5 mg/kg)	SA (10 mg/kg)	SA (20 mg/kg)
Initial body weight (g)	22.34 ± 1.54^###^	42.78 ± 2.09	42.68 ± 4.42	43.06 ± 3.47	40.28 ± 2.40	40.95 ± 2.94
Final body weight (g)	27.40 ± 1.74^###^	49.56 ± 2.7^###^	37.65 ± 6.48**	47.08 ± 7.53	51.51 ± 8.95	52.37 ± 8.28
Weight gain (g/day)	0.09 ± 0.02	0.12 ± 0.03	–0.09 ± 0.16**	0.07 ± 0.12	0.20 ± 0.16	0.20 ± 0.16
Food intake (g/day)	3.63 ± 0.45^###^	6.71 ± 1.31	5.99 ± 1.03	6.35 ± 0.74	6.93 ± 1.22	5.99 ± 0.98
Water intake (mL/day)	4.49 ± 0.35^###^	13.45 ± 0.75	12.20 ± 1.36	12.75 ± 1.33	12.62 ± 1.63	12.39 ± 1.29
FER (%)^b^	2.52 ± 0.58	1.84 ± 0.59	–1.62 ± 2.84**	1.07 ± 1.85	2.60 ± 1.86	3.33 ± 2.72

a Abbreviations: MET, metformin; FER, feed efficiency ratio. Results are expressed as the mean ± SD (*n* = 10). *Significantly different with respect to the db/m mice: *p < 0.05, **p < 0.01, and ***p < 0.001. ^#^Significantly different with respect to db/db mice: ^#^*P* < 0.005, ^##^*P* < 0.01, and ^###^*P* < 0.001.

b Feed efficiency ratio (%) = (body weight gain [g/day]/food intake [g/day]) × 100.

### Effect of SA on Renal Dysfunction in db/db Mice

**Figure 4:**
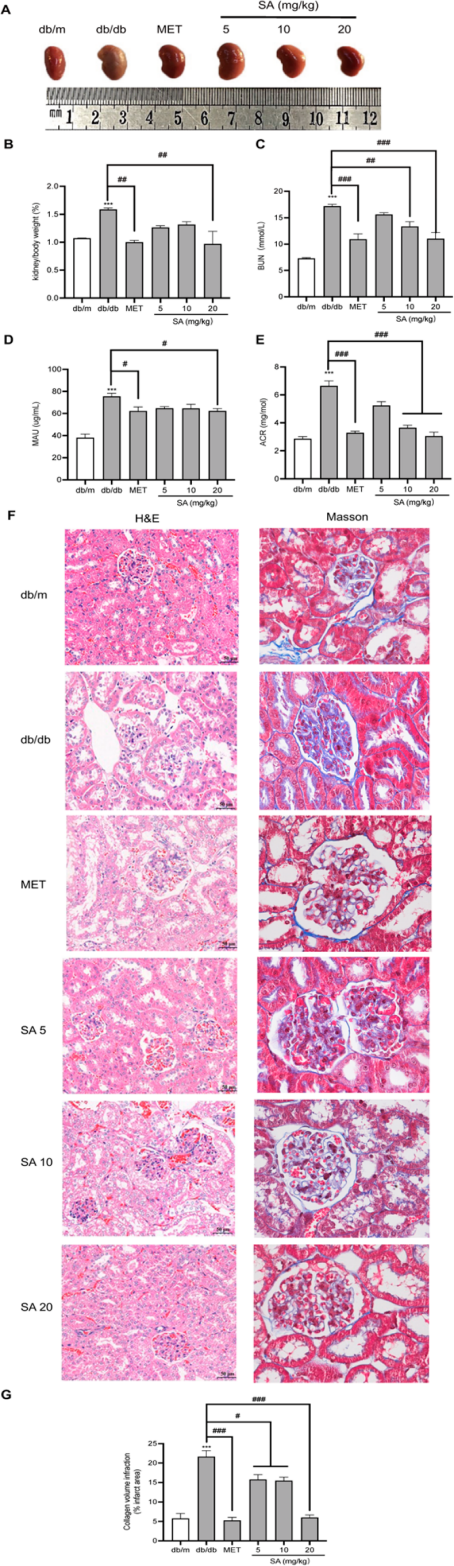
**Effects of SA on kidney structure and function in db/db mice.** (A) Representative pictures of the right kidneys from each group. (B) Ratio of kidney weight/body weight. (C–E) Serum levels of BUN, MAU and ACR in db/db mice (*n* = 8). (F) H&E staining (100× magnification) and Masson staining (400× magnification) showed the pathological changes in the kidneys of db/db mice. db/m and db/db, normal saline; MET, MET 200 mg/kg; SA 5, SA 5 mg/kg; SA 10, SA 10 mg/kg; SA 20, SA 20 mg/kg. (G) Collagen volume fraction quantified from the stained sections. All data are represented as the mean ± SEM. *p < 0.05, **p < 0.01, and ***p < 0.001 compared with the db/m group; ^#^*p* < 0.05, ^##^*p* < 0.01, and ^###^*p* < 0.001 compared with the db/db mice.

The kidney weight and ratio of kidney and body weight were recorded and calculated among the five groups in this research. As shown in Figure 4A, the size of the kidney in the db/db mice was significantly increased, which can be seen by appearance. Meanwhile, the db/db mice displayed a significant increase in the ratio of kidney and body weight. More importantly, the effect of SA was found to be statistically significant, as the ratio of kidney weight to body weight in the high-dose SA (20 mg/kg)-treated group returned to that of the MET group (Figure 4B). Renal fibrosis is closely related to its function. As shown in Figure 4C–E, the blood urea nitrogen (BUN), urinary microalbumin excretion (MAU), and albumin-to-creatinine ratio (ACR) levels were increased in db/db mice. However, SA treatment ameliorated these parameter changes, demonstrating that SA can protect renal function in db/db mice. Kidney structure abnormalities are the major cause of renal dysfunction. As shown in Figure 4F, H&E staining showed that db/db mice had notable glomerular hypertrophy, mesangial matrix expansion, glomerular hyaline degeneration, and tubulointerstitial fibrosis. Importantly, SA treatment at all the dosages markedly ameliorated those changes. Masson staining revealed collagen fiber deposition in the glomerular and renal tubules of db/db mice, and kidney fibrosis was significantly inhibited after SA treatment (Figure 4F,G). All the above-mentioned results indicate that SA has a protective effect on renal dysfunction in db/db mice.

### SA Suppressed TGF-β1/Smads Signaling in the Renal Tissues

**Figure 5:**
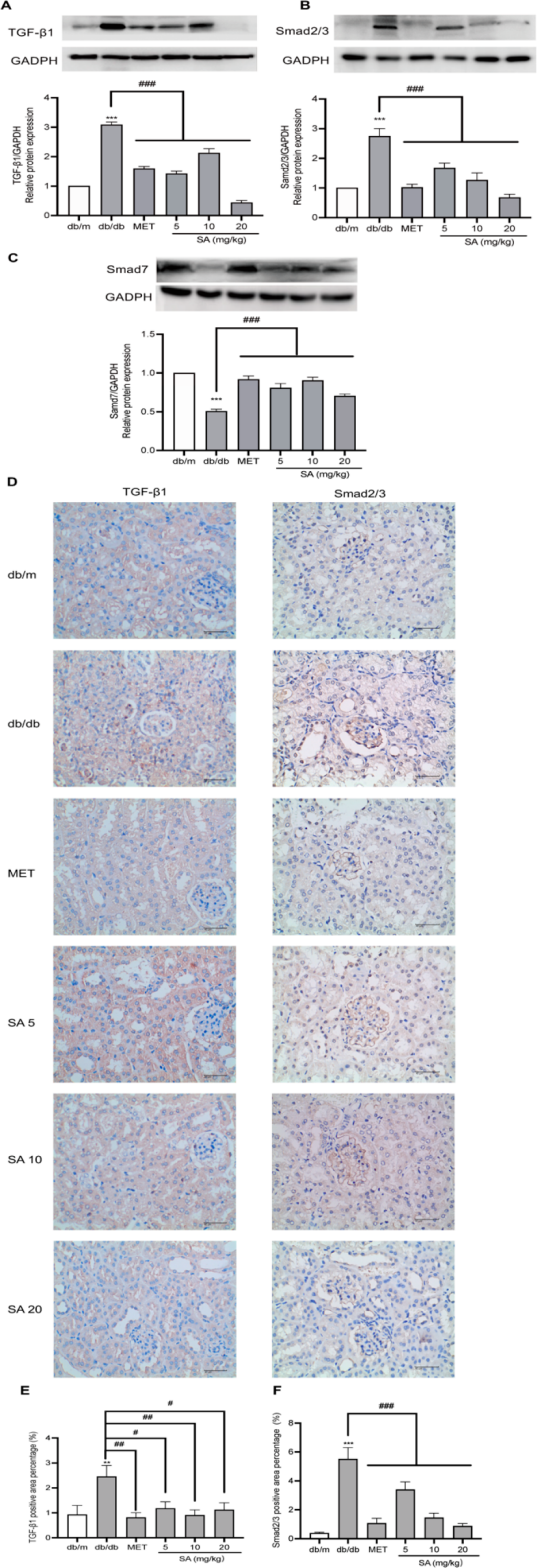
**SA regulated the TGF-β1/Smads signaling in the renal tissue** (A) Representative Western blot of TGF-β1 in kidney tissues and quantitative analysis of band intensity. (B) Representative Western blot of Smad2/3 in kidney tissues and quantitative analysis of band intensity. (C) Representative Western blot of Smad7 in kidney tissues and quantitative analysis of band intensity. (D) Representative immunohistochemistry images of TGF-β1 and Smad2/3 in the kidney tissues. db/m and db/db, normal saline; MET, MET 200 mg/kg; SA 5, SA 5 mg/kg; SA 10, SA 10 mg/kg; SA 20, SA 20 mg/kg. (E, F) Mean density of TGF-β1 and Smad2/3. All data are represented as the mean ± SEM (*n* = 3). *p < 0.05, **p < 0.01, and ***p < 0.001 compared with the db/m group; ^#^*p* < 0.05, ^##^*p* < 0.01, and ^###^*p* < 0.001 compared with the db/db mice.

The activation of the Smads pathway and its subsequent nuclear translocation are critical steps in TGF-β1-mediated fibrosis.^18^ In our study, the protein expression of TGF-β1 and Smads in kidney tissues was detected via Western blot (Figure 5). When compared with db/m mice, as expected, the protein expression of TGF-β1 and Smad2/3 was significantly increased and that of Smad7 was decreased in the db/db mice (Figure 5A–C). SA treatment of 10 mg/kg significantly increased the protein level of TGF-β1 compared to 5 mg/kg SA treatment when compared with db/db mice, while 20 mg/kg SA treatment tended to decrease the increase in protein expression of TGF-β1. These results showed no concentration dependence of the three doses of SA in reducing the expression of TGF-β1. Fortunately, it is undeniable that all three concentrations of SA treatments significantly reduced the expression of TGF-β1 and Smad 2/3 proteins when compared to the db/db group. Meanwhile, the protein expression of Smad7 was also significantly increased by SA treatment at all doses when compared with db/db mice (*p* < 0.001). To further demonstrate the role of SA in regulating the TGF-β1/Smads signaling pathway, TGF-β1 and Smad2/3 protein expression in kidney tissues was also detected by immunohistochemical analysis (Figure 5D–F). When compared with the db/m group, TGF-β1 and Smad2/3 expressions in renal tissues were significantly upregulated in db/db mice (*p* < 0.001 and *p* < 0.01, respectively), and this influence was attenuated by SA treatment at all doses for 8 weeks when compared with db/db mice. In addition, MET treatment significantly reduced the expression of TGF-β1 and Smad2/3 in renal tissues when compared to db/db mice. Specifically, the significant effect of SA treatment at a high dose was noted to restore the expression of TGF-β1 and Smad2/3 to that of the MET group. The above results show that SA downregulates TGF-β1/Smads signaling to mitigate renal fibrosis.

## Discussion

Fibrosis is the ultimate pathological feature of many chronic diseases, leading to considerable morbidity and mortality, which leads to an interest in finding treatments to effectively reverse the disease.^19^ In our study, ISO was used to induce cardiac fibrosis, and oral administration of SA was used to treat animals for 14 days to assess its anti-fibrosis effect. H&E staining and Masson’s trichrome staining showed disordered myocardial cells and deposition of myocardial interstitial and perivascular collagen in the ISO group. The above pathological changes were improved to varying degrees after SA intervention. Myocardial fibrosis is a complex chain of dynamic processes in which inflammation is a key factor in the myocardial remodeling process.^20^ Recent evidence has shown that some proinflammatory cytokines are involved in the development and progression of tissue fibrosis. Among these, IL-6 has been shown to be involved in cardiac dysfunction caused by ISO.^21^ TNF-α promotes the proliferation of cardiac fibroblasts *in vitro* and regulates collagen production through extracellular regulation of protein kinase signaling.^22^ We observed that SA significantly reduced the release of proinflammatory cytokines (IL-6, TNF-α, and IL-1β) in our research.

Moreover, it is well known that the activation of cardiac fibroblasts transforms them into myofibroblasts and produces ECM proteins.^23^ The ECM consists mainly of collagen I and III, the excessive accumulation of which then leads to structural and functional abnormalities in the heart.^24^ In our study, we also observed a significant upregulation of α-SMA, type I collagen, and type III collagen mRNA expression, suggesting that cardiac fibroblasts have differentiated into myofibroblasts, leading to an increase in collagen synthesis. In previous studies, oral administration of the precursor compound of SA (LS-102) produced a pharmacokinetic profile different from that of AS-IV, with higher bioavailability roughly about twice that of the prototype and toxicity tolerability similar to previous estimates.^25^ Notably, our results showed that SA treatment for 14 days downregulated α-SMA mRNA expression and reduced collagen accumulation, especially collagen I and III, confirming the potential value of SA in the treatment of cardiac fibrosis. MMPs are essential for the degradation of the ECM. Their activity directly affects the amount of the ECM and the function and structure of the heart. Increased expression of MMP-9 and MMP-2 leads to collagen fibrosis through excessive degradation of collagen.^26^ It is worth noting that SA treatment effectively inhibited fibrosis by suppressing the mRNA expression of MMP-2/9 and TIMP-1/2. The above results suggest that SA ameliorated cardiac fibrosis by reducing collagen accumulation and fibrosis-related inflammatory signals, including TNF-α, IL-1β, and IL-6.

The db/db mice are the most common model of type 2 diabetes.^27^ These models develop excessive obesity with persistent hyperglycemia and hyperlipidemia. Numerous experimental studies have shown that glomerular fibrosis is one of the main features of renal histopathology in late-stage db/db mice.^28^ In our study, to further provide the availability of SA and investigate the anti-fibrotic effects of long-term oral administration of SA, db/db mice presenting diabetic renal fibrosis were used, increasing the duration of oral administration (8 weeks) as well as using multiple dosing concentrations. Our study indicated that oral administration of SA has no significant hypoglycemic or body weight-lowering effects in db/db mice, which is consistent with other experimental results.^29^ Interestingly, evidence accumulated from many reports showed that dyslipidemia, specifically cholesterol-rich lipoproteins, plays a key role in the progression of kidney disease in patients with diabetes. TC, TG, LDL, and HDL are important indicators of blood lipid analysis.^30^ In our study, as expected, both TC and TG levels were significantly elevated in the db/db group and significantly downregulated by SA treatment for 8 weeks. Additionally, TG is the main energy storage unit in the body and is an important source of energy for daily activities. High concentrations of TG in serum may lead to excessive accumulation of hepatocytes, possibly resulting in hepatic steatosis.^31^ Interestingly, hepatic steatosis was also found in our H&E staining of the livers in db/db mice. However, lipid deposition in the liver was significantly decreased after SA treatment, indicating that SA conferred its protective role by ameliorating lipid metabolism impairment in db/db mice.

Renal enlargement is one of the typical features of structural disorders of the kidneys.^32^ In the development of kidney fibrosis, SA significantly reduced kidney weight and the ratio of kidney weight to body weight. Most importantly, pathological analysis of the kidneys showed that SA significantly ameliorated the pathological changes in the kidneys and the deposition of collagen fibers. In addition, kidney structure abnormalities are the major causes of renal dysfunction. Therefore, some of the biochemical parameters significantly related to renal function, such as BUN, MAU, and ACR, were also measured and found to be significantly reduced after SA treatment, suggesting that SA treatment prevented renal fibrosis and, thus, improved renal dysfunction in db/db mice.

TGF-β1 is one of the key factors leading to tissue fibrosis. It can be activated in response to inflammatory and mechanical conditions and confers on myofibroblasts the ability to proliferate, migrate, and synthesize collagen following activation.^33^ In our current study, Western blot and immunohistochemical analyses were both used to assess that the protein levels TGF-β1 and Smad 2/3 were significantly inhibited by SA treatment at three dosages, but molecular docking demonstrated that SA did not directly inhibit TGF-β1, as its binding affinity was only −9.3 kcal/mol; these results are described in detail in the Supporting Information, Figure S1. Local tissue hypoxia occurs during the formation of tissue fibrosis, where hypoxia-inducible factor-1α (HIF-1α) is one of the main cellular responses to hypoxia, allowing cells to adapt to hypoxic conditions by the expression of hundreds of genetic targets.^34^ Evidence has shown that increased protein levels of HIF-1α might activate the TGF-β1/Smads pathway and thus promote collagen deposition in keloid tissue.^35^ HIF-1α has also been reported to further synergistically enhance the fibrogenic effects of TGF-β1 by affecting the Smads pathway.^36^ Most importantly, AS-IV was reported to reduce HIF-1α expression and enhance Smad7 expression, thereby reducing kidney injury and preventing kidney fibrosis.^36,ref38^ In the present study, although we demonstrated that SA could affect the TGF-β1/Smads signaling pathway to improve tissue fibrosis, SA did not directly inhibit TGF-β1. We, therefore, speculated that SA might act on HIF-1α, an upstream target of TGF-β1, to reduce fibrosis in the heart and kidney. Unfortunately, modulation of the upstream target of TGF-β1 (HIF-1α) by SA was not revealed in this study, which was a limitation of this study, and this will be the focus of our future work. Exploring this issue might provide new insights into the effect of the long-term oral administration of SA on cardiac and renal fibrosis.

## Conclusions

In summary, our findings reflect the efficacy of long-term oral administration of SA that ameliorated cardiac and renal fibrogenesis. SA effectively induces beneficial changes at the histopathological or functional level in the kidney and heart, and the underlying mechanisms may regulate the activation of the TGF-β1/Smads signaling pathway. The data obtained from this study may lead to the oral-route medication availability of SA, thus offering a novel lead compound for developing long-term therapy to treat and prevent fibrosis.

## Materials and Methods

### Materials

Tris(2-carboxyethyl)phosphine (TCEP), *N,N,N′,N′*-tetramethylethylenediamine (TEMED), and ammonium persulfate (APS) were purchased from Sigma-Aldrich Co., Ltd. (St. Louis, MO, USA); 30% acrylamide, bis solution, and nonfat milk powder were purchased from Solarbio Science & Technology Co., Ltd. (Beijing, China); anti-TGF-β1 was purchased from Bioss Co., Ltd. (Beijing, China); anti-Smad2/3, anti-Smad7, and goat anti-rabbit IgG were purchased from Solarbio Science & Technology Co., Ltd. (Beijing, China); TRIzol Reagent was purchased from Invitrogen (California, USA); Transcriptor First Strand cDNA Synthesis Kit was purchased from F. Hoffmann-La Roche, Ltd. (Switzerland, Germany); NovoStart SYBR qPCR SuperMix Plus was purchased from Novoprotein Scientific Inc. (Shanghai, China); all primers were purchased from Sangon Biotech. Co., Ltd. (Shanghai, China); RAT IL-6, IL-1β, and TNF-α ELISA kits were purchased from Multi science (Lianke) Biotech Co., Ltd. (Hangzhou, China); serum triglyceride (TG), total cholesterol (TC), low-density lipoprotein (LDL), high-density lipoprotein (HDL), blood urea nitrogen (BUN), albumin-to-creatinine ratio (ACR), and urinary microalbumin (MAU) excretion assay kits were purchased from Shanghai Enzyme-linked Biotechnology Co., Ltd. (Shanghai, China); and isoproterenol (ISO) and dichlorodimethylsilane were purchased from Shanghai Aladdin Bio-Chem Technology Co., Ltd. (Shanghai, China). All the chemicals used in this research were analytical reagents and were purchased locally.

### Synthesis of SA

**Figure 6:**
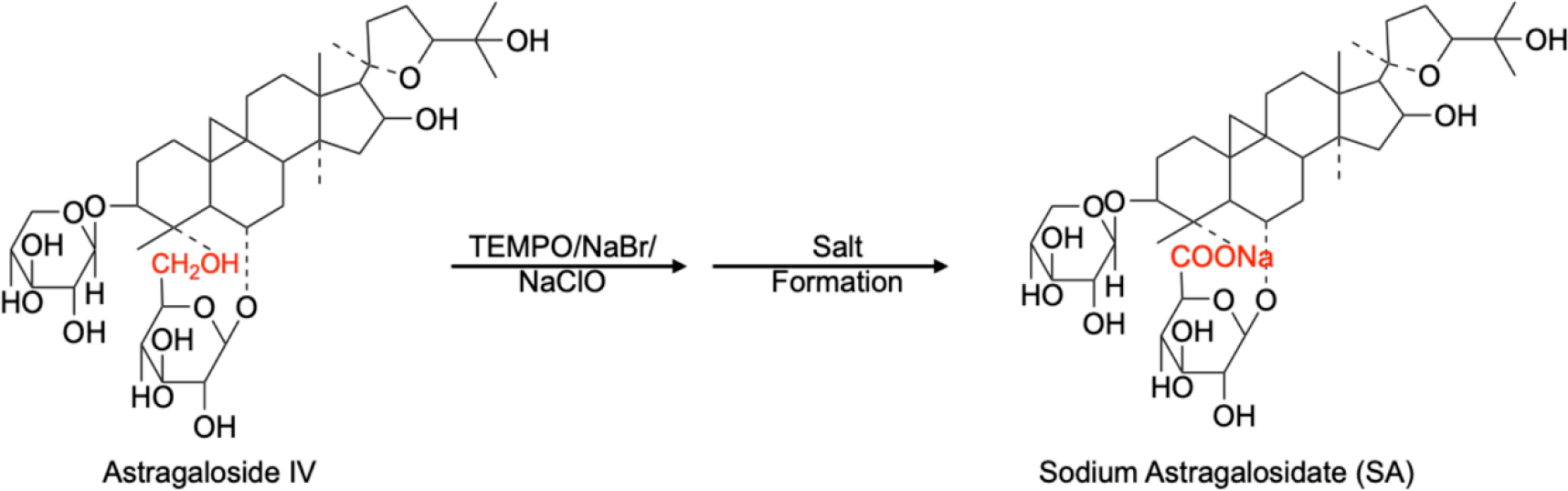
Illustration of the derivative synthesis of SA.

First, 5.0 g of AS-IV, 0.5 g of sodium bicarbonate, 0.2 g of 2,2,6,6-tetramethyl-1-piperidinyloxy, 1.35 g of anhydrous sodium carbonate, and 0.5 g of sodium bromide were added to 300 mL of 50% THF/H_2_O solution. After complete dissolution by stirring at 0 °C, sodium hypochlorite aqueous solution was added dropwise into the reaction solution until the reaction was complete. The THF solvent was removed, and crude SA was obtained by filtration. Finally, the sample was washed with 10% sodium formate aqueous solution three times and dried under reduced pressure to obtain 3.3 g of pure SA (Figure 6). The NMR data of SA are given in the Supporting Information, Figure S2.

### Animals

Male B6/BKS(D)-Lepr^db^/J (db/db) mice, non-diabetic (db/m) littermates, and male Sprague–Dawley (SD) rats were purchased from Jiangsu GemPharmatech Biotechnology Company (Jiangsu, China). The animals were housed in the stable environment of an animal room in a specific pathogen-free (SPF) laboratory under room temperature (20–23 °C) and humidity (50%–60%) conditions with standard light (12 h light/dark cycle). The animals were given free access to water and a normal diet. The study was approved by the Animal Ethics Committee of the State Key Laboratories for Quality Research in Chinese Medicines, Macau University of Science and Technology.

### Experimental Design and Drug Treatment

Thirty SD rats were randomized into three groups, i.e., control, ISO, and SA (20 mg/kg), and each group comprised 10 animals. As shown in Figure 1A, myocardial fibrosis rat model was established by referring to the literature; except for the control group, all the rats were injected with isoproterenol (ISO, 100 mg/kg/day) intraperitoneally for two consecutive days.^15^ The control group was injected with the same volume of normal saline. After modeling, all the rats were fed normally for 49 days. After that, SA (20 mg/kg) was orally administered once daily for 14 days. Meanwhile, the rats in the control group and the ISO group were given the same volume of normal saline. After the last dosing, the rats were anaesthetized using isoflurane, and all blood samples were collected to clot at room temperature for 1 h and then centrifuged at 2500 rpm for 10 min to obtain the serum. Then the rats were sacrificed to collect the heart. The interleukin 6 (IL-6), interleukin 1 beta (IL-1β), and tumor necrosis factor α (TNF-α) expressions were detected in serum by ELISA kits. All the samples were frozen at −80 °C.

The db/db mice were randomized into five groups, i.e., the db/db group, the metformin group (200 mg/kg, MET), and the SA group (5, 10, and 20 mg/kg, SA), and each group comprised 10 animals. Ten db/m mice were used as the control group (db/m). Briefly, as shown in Figure 3A, SA (5, 10, and 20) mg/kg/day was orally administrated for 8 consecutive weeks. MET was also orally administered once daily for 8 weeks in the MET group. Normal saline was given to the db/m and db/db groups. All mice had free access to food and water during the experimental period. The fasting blood glucose levels and body weight in each group were recorded once a week. After the last dosing, the mice were housed in individual metabolic cages to collect 24 h urine samples to assess the levels of BUN, MAU, and ACR. At the end of the experiment, mice were anaesthetized using isoflurane, and all blood samples were collected to detect the TC, TG, LDL, and HDL levels in serum. Finally, mice were sacrificed to collect the kidneys and liver. All the samples were frozen at −80 °C until the next use.

### Histopathological Examination

The kidney, liver, and heart tissues were fixed in 10% formalin and embedded in paraffin to prepare for hematoxylin–eosin (H&E) and Masson’s trichrome staining. After deparaffinization and rehydration, 5 μm thick sections were stained with H&E to evaluate tissue structural injury, and Masson’s trichrome staining was performed to observe the degree of tissue fibrosis. Finally, the sections were observed and photographed with an optical microscope. Each sample slice was observed under a microscope (Pannoramic 250, 3DHISTECH, Jinan, China) at a magnification of 100× or 400×.

### Gene Expression Analysis

Total RNA from the heart tissue samples was isolated using TRIzol reagent according to the manufacturer’s manual. Then, the RNA was reverse transcribed into complementary DNA using the Transcriptor First Strand cDNA Synthesis Kit. The specific primers for the heart tissue samples are listed in Table 2. qRT-PCR was performed in 10 μL reactions using PerfectStart Green qPCR SuperMix (+Dye I/+Dye II) and was calculated using the 2^–ΔΔCt^ method. The housekeeping gene GAPDH was used to normalize the gene mRNA expression.

**Table 2:** Specific Primer Information

Primer	Sequence (5′–3′)
β-Actin Forward Primer	AGCCATGTACGTAGCCATCC
β-Actin Reverse Primer	CTCTCAGCTGTGGTGGTGAA
MMP-2 Forward Primer	TCCAATGATGACATCAAGGGG
MMP-2 Reverse Primer	GTCCGCCAAATAAACCGATC
MMP-9 Forward Primer	GTACAGCCTGTTTCTGGTGGC
MMP-9 Reverse Primer	GGCCTTGGGTCAGGTTTAGAG
Collagen I Forward Primer	CCGTGACCTCAAGATGTGCC
Collagen I Forward Primer	GAACCTTCGCTTCCATACTCG
Collagen III Forward Primer	GACCTCCTGGAAAAGATGGATC
Collagen III Forward Primer	AAATCCATTGGATCATCCCC
α-SMA Forward Primer	TTATTGCTCCTCCAGAAC
α-SMA Reverse Primer	CTTCGTCATACTCCTGTT
TIMP 2 Forward Primer	CTTCATATTTCTTCCTTCCT
TIMP 2 Reverse Primer	GAACCTTGAGAGTGATTAC
TIMP 1 Forward Primer	TCTGGCATCCTCTTGTTG
TIMP 1 Reverse Primer	GCTGGTATAAGGTGGTCTC

### Western Blot

The kidneys from each group were homogenized in ice-cold RIPA lysis buffer, and the tissue lysates were centrifuged for 15 min at 8000*g* at 4 °C. The BCA Protein assay kit was used to detect the concentration of protein in the upper layer of the solution. Equivalent amounts of protein (30 μg) were separated by 10% SDS-PAGE gels and then transferred by electroblotting to the PVDF membranes (GE Healthcare Life Sciences, USA). The membranes were blocked in 5% (w/v) nonfat milk at room temperature for 1 h, after which they were incubated overnight at 4 °C with primary antibodies against TGF-β1 (1:1,000), anti-Smad2/3 (1:1000), anti-Smad7 (1:1000), and GAPDH (1:1000). The membranes were washed in TBST buffer three times and then incubated with secondary antibody (1:1000) at 37 °C for 1 h. Finally, the PVDF membrane was washed with PBST three times and then scanned by Bio-Rad Image Lab Software. GAPDH was used to normalize the target protein expression. ImageJ software was used to determine the intensity of the protein.

### Immunohistochemistry Analysis

Immunohistochemical staining of the kidneys was performed as described in previous studies. Briefly, paraffin-embedded kidneys were cut into 6 μm sections, dewaxed, and rehydrated. After 20 min of antigen retrieval in 10 mM sodium citrate (pH 6.0), the sections were incubated with 3% hydrogen peroxide for 10 min at room temperature and then blocked with 10% goat serum for 1 h at 37 °C. The sections were incubated with primary antibodies, including anti-TGF-β1 and anti-Smad2/3, overnight at 4 °C. The slides were washed with TBST and then incubated with anti-mouse/rabbit IgG complex for 1 h at room temperature. Finally, the sections were washed with TBST three times, and antibody binding was detected using 3,3′-diaminobenzidine tetrahydrochloride (DAB). Slides were counterstained with a hematoxylin solution. Finally, an Olympus DP72 microscope (Olympus Corporation, Tokyo, Japan) was used to view the slides and take images with a high-resolution camera at magnifications of 400× and 100×, respectively. The percentage of positive area was quantified by using ImageJ version 1.6.0_24 (National Institutes of Health, Bethesda, MD, USA).

### Statistical Analysis

Data are presented as the mean ± SEM of at least three repeated experiments. Statistical analyses were accomplished with GraphPad Prism 8.0 (GraphPad Software, San Diego, CA, USA) and performed using Student’s *t* test. The differences among multiple groups were evaluated by a one-way analysis. All experiments were performed at least in triplicate. *P*-value < 0.05 was considered statistically significant.

## Supporting Information Available

Figure S1, molecular docking of SA with TGF-β1 enzyme (PDB code: 6GFF); Figure S2, NMR data of SA; Table S1, interacting amino acids, H-bonds distance, and binding scores of TGF-β1 enzyme with SA (PDF)
