# Supplementary material for: Preclinical Investigations on Anti-fibrotic Potential of Long-Term Oral Therapy of Sodium Astragalosidate in Animal Models of Cardiac and Renal Fibrosis
Source: ACS Pharmacol Transl Sci. 2024 Jan 12;7(2):421–31. doi: 10.1021/acsptsci.3c00264 (PMC10863439; doi:10.1021/acsptsci.3c00264)
Supplement: Supplementary file 1 — pt3c00264_si_001.pdf [file pt3c00264_si_001.pdf]

Supplementary Materials

Title: Preclinical Investigations on Anti-Fibrotic Potential of Long-Term Oral Therapy of Sodium Astragalosidate in animal models of Cardiac and Renal Fibrosis

Xiao-Yi Chen[1,4 ^], Tian-Tian Wang[1, 2, ^], Qing Shen[1,3, *], Hao Ma[1], Zhan Hua Li[1], Xi-Na Yu[1], Xiao-Feng Huang[2], Lin-Sen Qing[2, *], Pei Luo[1, *]

1 State Key Laboratories for Quality Research in Chinese Medicines, Faculty of Pharmacy, Macau University of Science and Technology, Macau, China

2 Chengdu Institute of Biology, Chinese Academy of Sciences, Chengdu, China

3 Collaborative Innovation Center of Seafood Deep Processing, Zhejiang Province Joint Key Laboratory of Aquatic Products Processing, Institute of Seafood, Zhejiang Gongshang University, Hangzhou, China

4 Institute of Medicinal Biotechnology, Chinese Academy of Medical Sciences, Peking Union Medical College, Beijing, China.

^ These authors contributed equally to this work.

*Correspondence: qingls@cib.ac.cn (Lin-Sen Qing.); pluo@must.edu.mo (Pei Luo); leonqshen@163.com (Qin Shen).

1. The Interaction of TGF-β1 Enzymes with SA via Molecular Docking.

The crystal structure of TGF-β1 enzyme with 3.1 Å resolution complex (PDB code: 6GFF) was obtained from the PDB database https://www.rcsb.org/. The 3D structure models of LS-102 and AS-IV were built and minimized by using the program ChemDraw 19.0, and then saved in mol2 format. As enzyme preparation, the ligands and water molecules were removed, then polar hydrogen atoms and Gasteiger charges were added by using AutoDockTools 1.56. Moreover, the docking input files of target proteins and ligands were prepared as PDBQT format by using the AutoDock Tools software. Finally, Molecular docking of compounds into the crystal structure of TGF-β1 enzyme (PDB code: 6GFF) were performed via AutoDock Vina 1.12 software by Lamarckian genetic algorithm, it was used for docking against the receptors and to estimate the binding affinities (kcal/mol). The Lamarckian genetic algorithm implemented in Auto Dock Vina was utilized as the key search protocol. There is a configuration file (config.txt) that the path to the receptors and ligands were set. The config.txt files contain the receptor file name, the ligand file name, the x, y, and z coordinate of the center of the grid box, the size of the grid box dimension and maximum number of bindings poses to be generated for each dock. The parameters were the following: a grid box was prepared individually for TGF-β1 to cover the pocket with the main residues for enzyme binding site by maintaining the grid size at 126 × 94 × 126 Å in the x-, y-, and z-axis, respectively, and a grid size of x = 20.0 y = 54.1 z = 4.4 Å for the TGF-β1, with 1 Å grid spacing for all the enzymes. Auto Dock Vina was run using an exhaustiveness value of 120, and the final number of conformations generated was set as 20. The ligands were individually evaluated in silico against TGF-β1 enzyme in triplicates and the average of the best conformation was chosen with the lowest docked energy, based on complete docking search. Hydrogen bond and bond length were used as parameters to measure the interaction between TGF-β1 and ligand in PyMOL software.

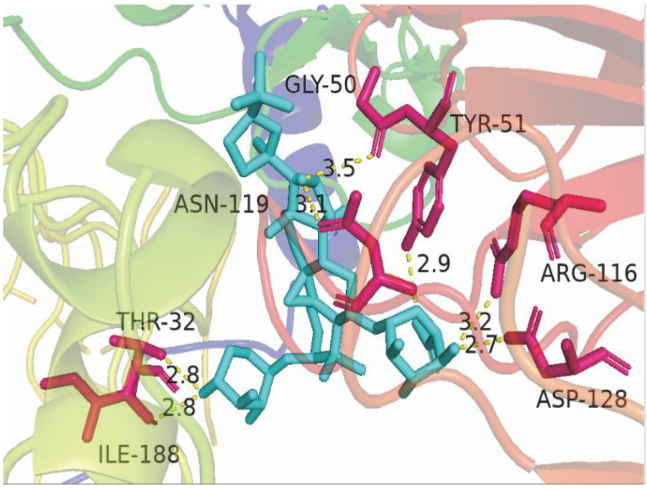

Fig. S1 Molecular docking SA with TGF-β1 enzyme (PDB code: 6GFF).

Table S1. Interacting amino acids, H-bonds distance, and binding scores of TGF-β1 enzyme with SA.

Name of the ligand	Binding Affinity (kcal/mol)	Number of hydrogen bonds	Distance (Å)	Residues
SA	-9.3	7	3.2	ARG-116
			2.7	ASP-128
			2.9	TYR-51
			2.8	ILE-188
			2.8	THR-32
			3.1	ASN-119
			3.5	GLY-50

2. Structure characterization of SA

As shown in **Fig. S2,** ^{13}C NMR data (150 MHz) of SA in D_2O (δ in ppm) were: 29.4 (C-1), 30.8 (C-2), 89.2 (C-3), 41.5 (C-4), 51.8 (C-5), 80.4 (C-6), 34.7 (C-7), 44.8 (C-8), 20.6 (C-9), 26.5 (C-10), 25.4 (C-11), 33.0 (C-12), 44.7 (C-13), 45.7 (C-14), 46.4 (C-15), 73.5 (C-16), 57.3 (C-17), 21.3 (C-18), 32.1 (C-19), 87.5 (C-20), 26.0 (C-21), 34.3 (C-22), 25.7 (C-23), 81.1 (C-24), 71.9 (C-25), 27.9 (C-26), 29.1 (C-27), 19.5 (C-28), 27.5 (C-29), 16.0 (C-30), 105.7 (C-1'), 73.8 (C-2'), 76.7 (C-3'), 71 (C-4'), 65.1 (C-

5'), 103.2 (C-1"), 73.6 (C-2"), 76.3 (C-3"), 72.1 (C-4"), 76.0 (C-5"), 175.5 (C-6").

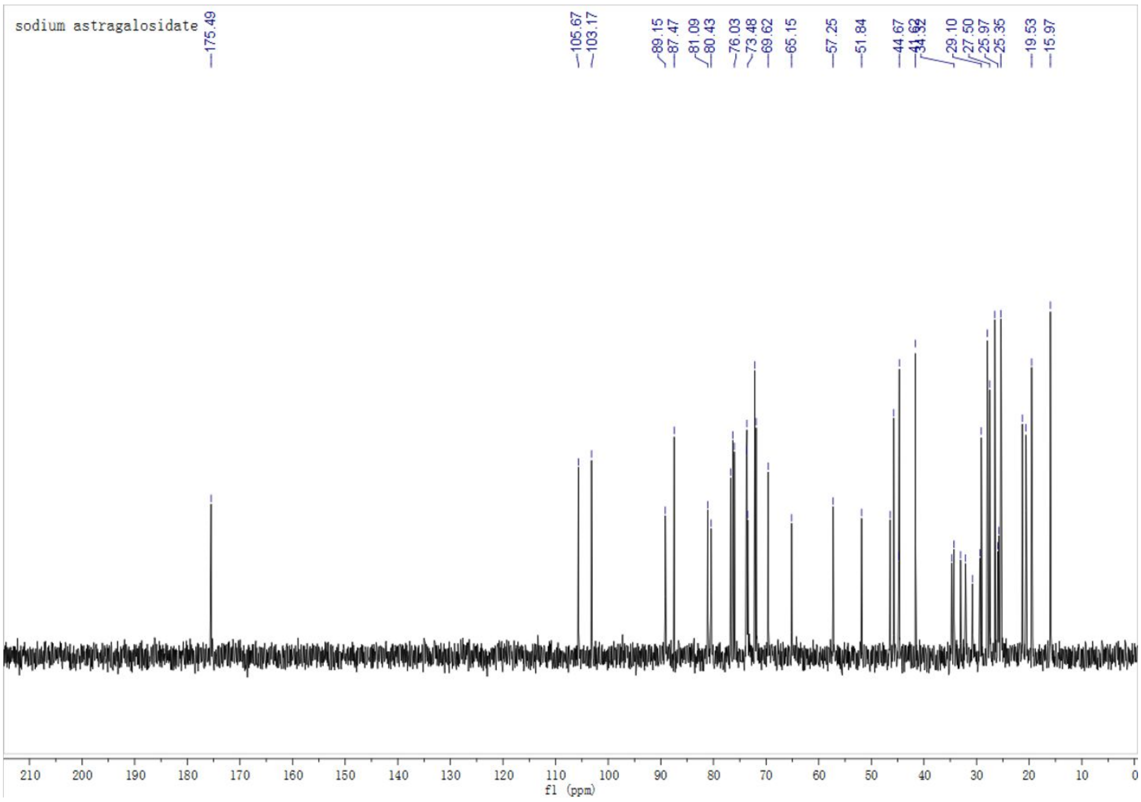

Fig. S2 The NMR data of SA.
